# Towards mature use of semantic resources for biomedical analyses

**DOI:** 10.1186/2041-1480-2-S5-I1

**Published:** 2011-10-06

**Authors:** D Rebholz-Schuhmann, F Rinaldi, S Pyysalo, N Collier, U Hahn

**Affiliations:** 1 EMBL Outstation, European Bioinformatics Institute, Hinxton, Cambridge, CB10 1SD, UK; 2 University of Zürich, Zürich, Switzerland; 3 Department of Computer Science, University of Tokyo, Tokyo, Japan; 4 National Institute of Informatics, 2-1-2 Hitotsubashi, Chiyoda-ku,Tokyo, Japan; 5 Language & Information Engineering (JULIE) Lab, Friedrich-Schiller-Universität, Jena, Germany

## 

Use of semantic resources, such as ontologies, has been the key to the standardisation of IT solutions and their interoperability in recent years. Combining semantic resources with biomedical data analysis is developing into a dedicated research domain and stimulates related research in biomedical research fields.

The growing attention to semantics-driven analyses motivates full-fledged conferences dedicated to the latest scientific results in this domain. The conference series entitled *Symposium on Semantic Mining in Biomedicine* (*SMBM*) constitutes a platform for activities of researchers showing interest in text and data mining in biomedicine, medical, bio- and chemo-informatics, and researchers from biomedical ontology design and engineering. The prospects of these meetings are the exchange of shared resources, the integration of existing resources and training in novel approaches and solutions.

The variety of scientific events focused on the generation of semantic resources and on the exploitation of semantic resources, including the biomedical literature and the Semantic Web, is testimony of the attention attributed to the field. Additionally, in recent years the progress in the use and analysis of semantic resources has been kept under constant assessment by demanding competitive evaluations such as BioCreative, CALBC and the BioNLP shared tasks [1-3]. This has led to the standardisation of semantic resources and their use for large-scale document processing [4].

Challenges remain, as demonstrated from the presented research work at the SMBM. Such challenges are the generation of consistent semantic resources, their formalisation and exploitation for biomedical analyses. Other challenges result from analyses of the whole scientific literature requiring the identification of relations and events at best in a large-scale approach and the identification of relevant contextual information, such as speculation and negation. The research work presented at the SMBM 2010 was addressing some of these challenges.

The transformation of the textual representation in the GENIA corpus into an axiomatic OWL representation was achieved by [5,6]. This solution improves the disambiguation of the names of genes and proteins in the GENIA corpus. The formal representation also enables consistency analysis across the corpus, as well as the development of verifiable annotation guidelines and the automatic discovery of new facts through deductive inferences.

Event extraction was addressed by [7] for the identification of DNA methylation events and by [8] for gene regulatory events. [9] identify semantic relations of medical entities, whereas [10] performed a large-scale analysis of the whole of Medline to collect and characterise associations of genes and proteins through syntactic parsing.

With the help of linguistic patterns and domain knowledge, [9] is linking the correct semantic relation to any pair of medical entities that have been identified from the scientific literature. For example, the assignment of a relation denoting the treatment of a disease could be achieved at 76% precision and 61% recall.

The identification of the DNA methylation events required the preparation of a specialised corpus to evaluate the performance of a retrained state-of-the-art event extraction system (78% precision, 76% recall). For the identification of gene regulatory events, [8] made use of a semantic resource for the events to derive inference rules encoding the domain knowledge. The inference has been applied to deduce implicit events from explicitly expressed events. The system has been evaluated against different gold standard data resources showing that the inference module contributes to 53.2% of the correct extractions, but does not cause any incorrect results.

At a larger scale, [10] screened all of PubMed to study statements of gene/protein associations based on a combination of solutions including syntactic parsing. The authors suggest an estimate for known event types from existing annotated resources in comparison to the requirements for the identification of such events types from PubMed: out of all event-type associations stated in PubMed that make reference to two proteins, over 90% may be of types for which annotated resources for event extraction exist.

Apart from the identification of entities, relations and events, other research work is concerned with the analysis of the discourse structure of the scientific literature, such as the resolution of co-reference [11] and the identification of speculation, negation and hedging [12,13].

The extraction of event-argument relations from the discourse in biomedical documents requires co-reference resolution to improve the recall of existing approaches. In the presented work by [11], an implementation based on Support Vector Machine (SVM) classifiers has been compared against a joint Markov Logic Network (MLN) and led to the finding that the latter outperforms the former.

For the identification of negation and speculation, [12] acquired hedge cues for the labelling of sentences according to certainty and as part of their solution applied random indexing to achieve dimensionality reduction necessary for large classifiers. [13] exploited linguistic cues from the BioScope corpus to categorize biological events in the GENIA corpus for uncertainty and negation.

Other research work presented at the SMBM is concerned with the processing of data streams [14,15] for the prediction of disease outbreaks and the generation of large-scale data resources [16].

[14] has trained a classifier to attribute class labels for avoidance behaviour, increased sanitation, seeking pharmaceutical intervention, wearing a mask and self-reported diagnosis to 5,283 Twitter messages. SVM classification performed better than a Naives Bayes classifier delivering evidence for a high degree of correlation between pre-diagnostic social media signals and gold standard laboratory data. The analysis of multilingual news for the prediction of disease outbreak leads to the result that sources in different languages improve the sensitivity of the prediction in comparison to English only news [15].

Another large-scale approach led to the generation of an annotated biomedical corpus (CALBC project) with four different semantic groups through the harmonisation of annotations from automatic text mining solutions, called the Silver Standard Corpus (SSC-I) [16]. This corpus has been used for the First CALBC Challenge asking the participants to annotate the corpus with their automatic annotators (named entity taggers). The best performance over all semantic groups was achieved from two annotation solutions that were trained on the SSC-I.

Altogether, the SMBM2010 symposium covered a large number of questions and tasks that reside on the edge of semantic resources, literature analysis and large-scale data processing. Growing all resources together could significantly mature the field of semantics in biomedicine. The key steps forward are determined by the standardisation, formalisation and reuse of semantic resources, i.e. ontologies in the first place, the stringent evaluation of analytical solutions and the interoperability of resources and analytical solutions on a large scale. Certainly SMBM will continue to contribute to these objectives in the future and will help to shape and judge on the progress.
